# Hyperparasitism in a Generalist Ectoparasitic Pupal Parasitoid, *Pachycrepoideus vindemmiae* (Hymenoptera: Pteromalidae), on Its Own Conspecifics: When the Lack of Resource Lead to Cannibalism

**DOI:** 10.1371/journal.pone.0124305

**Published:** 2015-04-24

**Authors:** Wei Chen, Zhang He, Xiao-Li Ji, Si-Ting Tang, Hao-Yuan Hu

**Affiliations:** Key Laboratory of Biotic Environment and Ecological Safety in Anhui Province, College of Life Sciences, Anhui Normal University, Wuhu, Anhui, P. R. China; CNRS, FRANCE

## Abstract

Hyperparasitism is a normal behavior of parasitoids, which often happens among species. Conspecific hyperparasitism, such as some kinds of heteronomous hyperparasitic behaviors, has been only reported in some species belonging to Aphelinidae. In this article, the conspecific hyperparasitism of *Pachycrepoideus vindemmiae* (Pteromalidae) is reported, with *Drosophila* puparia as hosts. Hosts were exposed to *P*. *vindemmiae* females twice to parasitism with nine, twelve, and fifteen day intervals between the two exposures. None of the infested hosts emerged more than one offspring, and emergence of parasitoid offspring occurred in two obvious events, synchronously with the exposure time intervals, which suggested that offspring emerging during the first and second events would come from the primary and secondary parasitoids, respectively, and the inference with the developmental duration of offspring also indicated this. With two *P*. *vindemmiae* strains that could be identified by a simple sequence repeat marker, the above speculation of the origin of those offspring emerging during the two events was confirmed. Dissection of hosts exposed twice revealed a cannibalism behavior of larvae from the secondary foundresses on the primary conspecific pupae. Our results suggested a conspecific hyperparasitism behavior of the secondary parasitoids on the primary conspecifics. Measures showed a reduced body size for the adults from the conspecific hyperparasitism. Foundresses from the conspecific hyperparasitism had less fitness variables than those from primary parasitism, with shorter longevity, less life time fecundity, lower values of infestation degree, and lower success rate of parasitism. However, when the parasitoids from the conspecific hyperparasitism met healthy *Drosophila* puparia, their offspring would recover to normal size. Frequency of the conspecific hyperparasitism behavior enhanced with the decreasing of proportion of healthy hosts in the oviposition patch. The conspecific hyperparasitism of *P*. *vindemmiae* on the primary conspecifics would be helpful to last the population when healthy hosts are absent in the oviposition patch.

## Introduction

Parasitoid wasps are insects whose larvae develop by feeding on the bodies of other arthropods, and larval feeding results in the death of the parasitoid’s host. The larvae’ development of parasitoid wasps totally depends on hosts. Sometimes, once hosts have been parasitized, the food chain becomes more complex because larvae of a secondary parasitoid can use primary parasitoids as food. Hyperparasitism happens when a secondary female of another parasitoid wasp species lays her eggs on the host, but the larvae of the secondary species feed on the primary parasitoids [1]. Two kinds of hyperparasitism exist, normally known as facultative and obligate hyperparasitism. The former happens when hyperparasitoids can attack healthy hosts and hyperparasitism occurs only when eggs are laid on a previously infested host. The latter occurs when hyperparasitoids can only develop on the primary parasitoid larvae or pupae. Generally, hyperparasitism often occurs among species, and multitrophic relationships exist in the food chains with several parasitoids, e.g. the system of pea aphid and parasitoid groups (reviewed in [2]).

Some special forms of hyperparasitism occur amongst conspecific species such as the Coccophaginae, a subfamily of Aphelinidae. The universally accepted heteronomous form of hyperparasitism occurs when some female parasitoids develop normally as primary endoparasitoids of primary hosts, whereas males develop hyperparasitically in primary endoparasitoids, including their own conspecific females [3–6].

Facultative hyperparasitoids can develop either as primary or as secondary parasitoids [2,7]. Previous studies have shown that *Pachycrepoideus vindemmiae* Rondani (Hymenoptera: Pteromalidae) is a facultative hyperparasitoid of *Drosophila* parasitoids [8–10]. Female *P*. *vindemmiae* can oviposit in puparium containing either a *D*. *melanogaster* or a primary parasitoid, including *Asobara tabida* Nees (Braconidae), *Fopius arisanus* (Sonan) (Braconidae), *Diachasmimorpha longicaudata* (Ashmead) (Braconidae), *D*. *kraussii* Fullaway (Braconidae), *Psyttalia concolor* (Szépligeti) (Braconidae), and *Leptopilina heterotoma* (Thomson) (Eucoilidae) [8–10]. Goubault et al. [11] pointed out that *P*. *vindemmiae* can superparasitize already infested hosts, in which the primary parasitoid were eggs or larvae even prepupal stages, when there is not enough available primary hosts. If the primary parasitoid is still a larva, the two larvae fight and the primary one mostly wins in competition for the host resource, and only one of the two parasitoids survives and emerges as adult stage, confirming the solitary status of *P*. *vindemmiae* [11]. If the primary parasitoid is already a prepupa or a pupa, the secondary parasitoid is forced to feed on the remains of the host, even if its final adult size is reduced, and the meeting between the pupa and the larva result into a coexistence with emergence of two parasitoids, firstly described as a gregarious status for *P*. *vindemmiae* [11]. Goubault *et al*. realized their experiments on *Delia radicum* (Diptera: Anthomyiidae) puparia as hosts, which would be large enough to receive and feed two parasitoids together [11]. However, if *P*. *vindemmiae* parasitize smaller hosts, like *Drosophila* puparia, we can emphasize that, if the parasitoid egg is laid in an already infested host, where the primary parasitoid is already a prepupa or a pupa, the secondary parasitoid larva will be forced to feed on the primary one, because of a lack of enough primary host resource. On this basis we can hypothesize that hyperparasitism behavior would occur in generalist ectoparasitic pupal parasitoids when (i) there are not enough available primary hosts, (ii) the primary parasitoids are at prepupal or pupal stage, and (iii) the hosts are too small to feed two parasitoids together.

To test this assumption, we obtained the prepupal or pupal stage of the primary *P*. *vindemmiae* parasitoid by exposing some *Drosophila melanogaster* (Diptera: Drosophilidae) puparia to the parasitoid at least nine days before, and exposed the already infested puparia to conspecific females with different time intervals between the two exposures. We used a new molecular primer to check for the origin of the emerged adults when two strains of *P*. *vindemmiae* have been used as the primary and secondary foundresses. Finally we measured different parameters of the primary and secondary foundresses’ offspring to discuss the relevance for *P*. *vindemmiae* females to adopt such a hyperparasitism behavior. To the best of our knowledge, this is the first report on this type of hyperparasitism behavior.

## Materials and Methods

*P*. *vindemmiae* is 2–3 mm long and usually solitary, often with one adult offspring emerging per host [12]. Hosts include the pupal stage of a range of cyclorrhaphous dipteran species [13,14]. The parasitoid is an ectoparasitoid, attacking puparia and laying eggs on the surface of the pupal body within the puparium wall. Newly hatched larvae consume the host, pupation continues inside the puparia, then adults emerge from the host’s puparium wall after larvae’s development [15].

### Origin and rearing conditions of the insects

Two strains of *P*. *vindemmiae* were used in this study. The Wuhu strain was obtained in May 2012 from the campus of Anhui Normal University, Wuhu, Anhui province, China, and the Urumqi strain was obtained in July 2012 from the campus of Xinjiang University, Urumqi, Xinjiang Uygur Autonomous Region, China. Both strains were maintained with puparia of *D*. *melanogaster* for about one year. Parasitoid wasps were reared in glass vials (25 mm in diameter by 50 mm in height) with *Drosophila* puparia and honey-soaked cotton wool as food, in an incubator with a set light:dark photoperiod (14:10). Temperature and relative humidity were 25 ± 1°C and 60 ± 5%, respectively. *Drosophila* puparia (aged less than two days) similar in size were given. Offspring emerging in 24 h were collected together for about 24 h to ensure that females had mated. *D*. *melanogaster* were collected in a Chinese bayberry orchard in Yuyao city, Zhejiang province, China, and cultured in the above described incubator on standard medium [16]. Adult fruit flies were reared in a cube cage made with nylon mesh of 150-μm pore size, and 90 mm petri dishes with medium were put into the cage. Every 12 h, the dishes were taken out of the cage and placed in a plastic box, about 50 L in volume, sealed with the nylon mesh around the rim. Four days later, the dishes were sprinkled with water and older *Drosophila* larvae crept into the box and pupation on the smooth box wall. The puparia were sprinkled and collected with a soft Chinese brush every day.

### Emerging times of offspring from *Drosophila* puparia exposed twice

If *P*. *vindemmiae* can use an already infested host, offspring would emerge later at an interval related to the time interval between the two exposures to parasitism. In order to test this, and also to compare offspring size, three treatments were designed, with nine, twelve, and fifteen day intervals between the two exposures. *P*. *vindemmiae* foundresses used were of the Wuhu strain, aged less than two days. The parasitoids were collected together into a 300 mL bottle, sealed with the nylon mesh, and honey-soaked cotton wool was provided as food. Puparia were less than two days old. Ten puparia were used as a group, and one female parasitoid was introduced as the primary foundress. 24 h later, the primary foundress was removed. Using the time intervals above, the secondary female parasitoids were placed and removed 24 h later. The puparia were placed individually and inspected daily until parasitoid offspring emerged. The foundresses and offspring were soaked into 75% alcohol before measurement. Ten groups were conducted for each treatment. In control treatment, ten puparia were given as a group, and the puparia were exposed only to the primary parasitoid for 24 h, and ten groups were conducted. The offspring number of both sexes and the emerging day was recorded. Hind tibia length has been used as a common measure of body size in parasitoids [1,17]. The hind tibia of offspring was removed from body and photographed with a digital microscope and the tibia length was measured.

### Discrimination between the respective offspring of the first and second emerging events

To check whether the later emerging offspring were derived from the primary or secondary foundresses, the Wuhu and Urumqi strains were used as primary and secondary foundresses, respectively. A simple sequence repeat (SSR) marker is used to identify the Wuhu and Urumqi strains (unpublished data). Ten puparia were collected together into the above bottle as a group, and exposed to one parasitoid female from the Wuhu strain for 24 h. Nine days after primary exposure, a secondary female parasitoid from the Urumqi strain was introduced in the bottle for another 24 h. The other experimental conditions were the same as described above. The exposed puparia were separately placed and checked daily to record the emerging day, and offspring were collected in 95% alcohol for identification. Twenty four groups were conducted in total. Genomic DNA was extracted from ethanol-preserved samples using the boiling method [18]. We used the genomic DNA for polymerase chain reaction (PCR), using the primer set BL8 (BL8, F:5′-CGTTTCTGTTTGTCATCGACAG; R:5′-AGATGGTTCGGCGATAAAGA). We identified the PCR products of the Wuhu strain containing the (AG)_10_ repeat, and Urumqi strains contained the (AG)_28_ repeat. All PCR products were detected by agarose gel electrophoresis. We analyzed the sequences of the PCR products using an ABI PRISM 3730 automated sequencer (Applied Biosystems, Foster City, CA, USA).

### Measurements of the primary and secondary foundresses’ offspring

To test the respective fitness of the primary and secondary foundresses’ offspring, two kinds of females were obtained, i.e. normal females and smaller ones from respectively primary and secondary parasitism. Two treatments were designed respectively with foundresses from primary parasitism and hyperparasitism on infested puparia exposed nine days before. And *P*. *vindemmiae* were of the Wuhu strain, aged less than two days. The parasitoids were collected together into a 300 mL bottle, sealed with the nylon mesh, and honey-soaked cotton wool was provided as food. Puparia were less than two days old. Ten puparia were used as a group, and one female parasitoid of the two types was introduced. Every 24 h, the ten exposed *Drosophila* puparia were changed with ten healthy puparia till the death of foundress. And the removed puparia were reared until parasitoid offspring emerged. Offspring were soaked into 75% alcohol for the measurement of hind tibia length with the method as represented above. Twenty groups were conducted for both treatments. In the treatment with females from hyperparasitism, 18 females had progeny of both sexes, but two females had only male progeny though out life time, which indicated that the females had not been mated, and data of those two groups were not analyzed.

We compared the values of degree of infestation (DI) and the success rate of parasitism (SP) between the two types of foundresses [19,20]. Numbers of adults *Drosophila* (di) and of parasitoids (pi) emerging from each group were counted. The DI measures the proportion of *Drosophila* killed by the parasitoid and is estimated as (T—di) / T, where T being the average number of emerging flies in the absence of the parasitoid. The SP measures the probability that an infested host will give rise to an adult wasp, and this is estimated as pi / (T—di) (if pi > (T—di), we set SP = 1).

### Hyperparasitic behavior on infested hosts provided with healthy hosts

To test whether the hyperparasitic behavior would happen when there are healthy hosts in the oviposition patch, both healthy and infested hosts were given together. Parasitoids from Wuhu and Urumqi strains were used as the primary and secondary parasitoids. Puparia which had exposed to the primary female parasitoid nine days before but without adult fly emergence were used as infested hosts. Three treatments were designed, with a ratio of healthy and infested puparia of 5:5, 3:7, and 1:9, respectively, in the given ten hosts. The mixed puparia were exposed to one secondary parasitoid female for 24 h, and reared until parasitoid adults emerged. The other experimental conditions were the same as described above. Six, eight, and six groups were conducted for the above three treatments, respectively. Emerged parasitoid adults were collected and the origin of the parasitoid offspring was discriminated with the SSR marker as described above.

### Statistic analyses

We used a linear model to analyze such normally distributed data as tibia size. But such count data as duration, longevity, number of offspring, and such proportion data as DI and SP often have non-normally distributed errors, and we used generalized linear model (GLM) analysis of deviance to those data, assuming Poisson errors and a log link function to count data, and binomial errors and a logit link function to proportion data. We assessed the appropriateness of the assumption of Poisson or binomial errors by comparing the residual deviance with the residual degrees of freedom after fitting the explanatory variables. Large relative values of the residual deviance indicate overdispersion, which may result in an overestimation of significance levels, and we replaced Poisson or binomial with quasipoisson or quasibinomial in the analyses. When more than one explanatory variable were considered, a full model was initially fitted to the data, including explanatory variables and their interactions. Terms were then removed from the full models by stepwise deletion. Whether the removal of a term caused a significant increase in deviance was assessed with a χ^2^ test. The final models were tested using an *F*-test [21]. All of the analyses were conducted in R2.13.0 [22].

## Results

### Developmental duration and body size of offspring from primary and secondary parasitism

**Fig 1:**
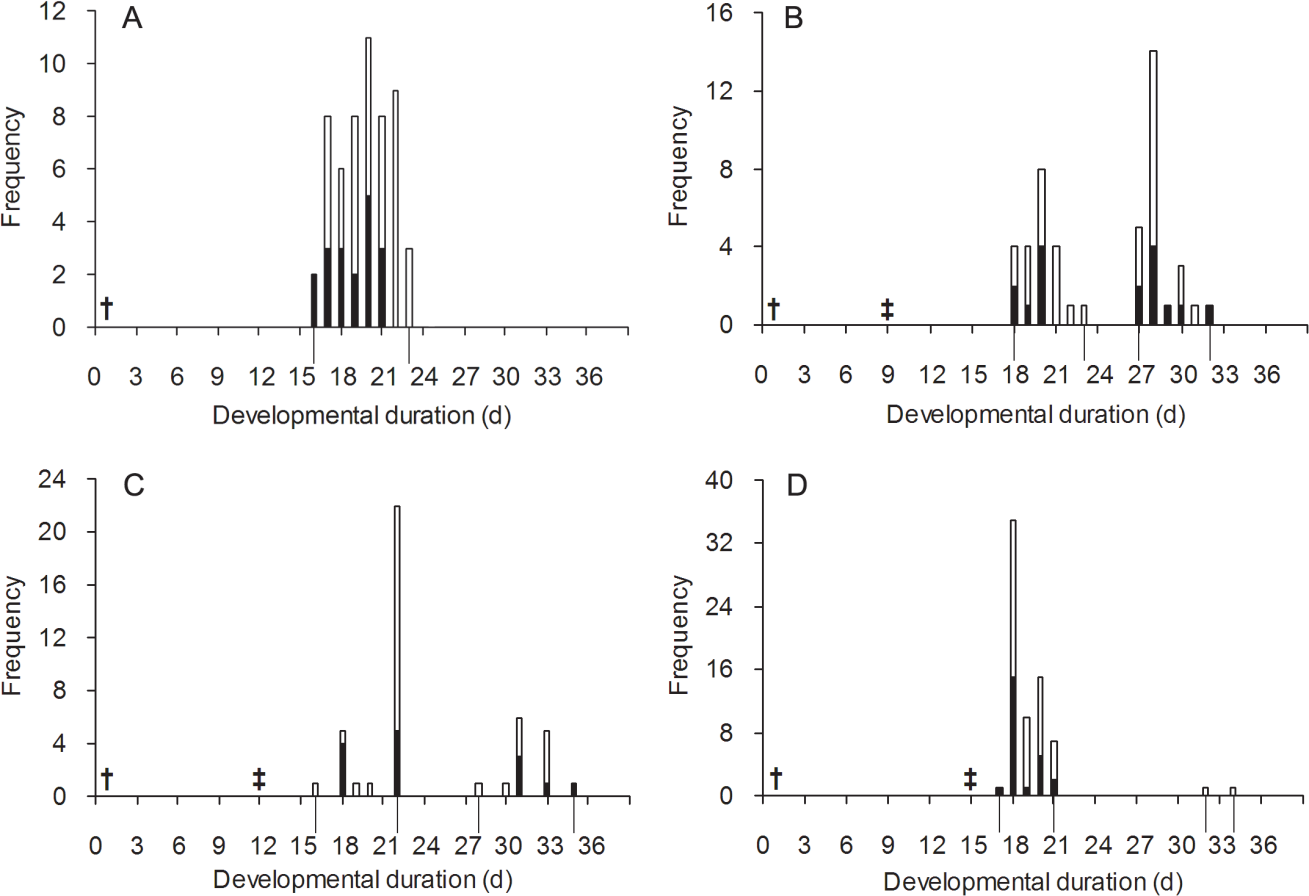
Frequencies of developmental duration of *P*. *vindemmiae* offspring. A: exposed only once; B-D: exposed twice with nine, twelve and fifteen day intervals. † and ‡ indicate primary and secondary exposure, respectively. Open and solid bars indicate females and males, respectively. Offspring emerged at two different events when the two exposures occurred with different time intervals.

In all of our experiments, just one *P*. *vindemmiae* parasitoid emerged per puparium. The parasitoid could engage in parasitic behavior on hosts already parasitized by conspecific females. The duration from primary exposure to parasitoid offspring emergence differed among the control and the three treatments with nine, twelve, and fifteen day intervals between the primary and secondary exposures (*F*_3, 212_ = 19.63, *P* < 0.01). And the duration also differed among the control and the two emerging events (*F*_1, 211_ = 92.89, *P* < 0.01). Offspring emerged at two different times when the two exposures occurred with different time intervals (Fig 1). Offspring of the second emerging event had significantly longer durations compared to both the first emerging event (with nine day interval: *F*_1, 45_ = 35.09, *P* < 0.01; with twelve day intervals: *F*_1, 42_ = 43.24, *P* < 0.01) and the control (with nine day interval: *F*_1, 78_ = 55.33, *P* < 0.01; with twelve day intervals: *F*_1, 67_ = 66.53, *P* < 0.01).

In the treatments with an interval of nine, twelve and fifteen day between primary and secondary exposures, there were eight, nine and two experimental groups emerging with the two events, and the time interval between means of the emerging events per group was 8.89 ± 1.49 (*N* = 8), 10.98 ± 2.83 (*N* = 9), and 13.53 ± 1.07 (*N* = 2) days, respectively, in the three treatments. The emerging time intervals were similar with the exposing time intervals in the treatments with an exposure interval of nine days (*t*_7_ = 0.20, *P* = 0.84), and twelve days (*t*_8_ = 1.08 *P* = 0.31), which suggested that offspring of the two emerging events would probably be those from the two exposures to parasitism. If the offspring of the first and second emerging events were taken as the primary and secondary parasitoids, respectively, then there were no significant differences for the offspring developmental duration among the control and the three treatments (*F*_3, 212_ = 1.36, *P* = 0.25), and no significant differences among the control and the two emerging events (*F*_1, 211_ = 0.83, *P* = 0.36) (Table 1). When both sexes were analyzed separately, developmental duration of female offspring was similar among the control and the three treatments (*F*_3, 140_ = 1.07, *P* = 0.36), and no significant differences among the control and the two emerging events (*F*_1, 139_ = 1.30, *P* = 0.25). And that of male offspring was also similar among the control and the three treatments (*F*_3, 68_ = 0.44, *P* = 0.72), and no significant differences among the control and the two emerging events (*F*_1, 67_ = 0.01, *P* = 0.93) (Table 2).

**Table 1:** Developmental duration and size of *P*. *vindemmiae* offspring at the first and second emerging events from hosts exposed twice with different intervals.

Interval (d)	Offspring number		Duration (d)		Tibia length (mm)	
	First	Second	First	Second	First	Second
9	22	25	19.86 ± 1.32	19.36 ± 1.29	0.33 ± 0.02^a^	0.28 ± 0.03^b^
12	30	14	20.97 ± 1.83	19.71 ± 1.73	0.34 ± 0.02^a^	0.26 ± 0.03^b^
15	68	2	18.88 ± 1.10	18.00 ± 1.41	0.35 ± 0.02	0.33 ± 0.01
ck	55	—	19.69 ± 1.93	—	0.32 ± 0.02	—

Different letters means significant differences between the first and second emerging events. The same below.

**Table 2:** Developmental duration and size of female and male *P*. *vindemmiae* offspring at the first and second emerging events from hosts exposed twice with different intervals.

Interval (d)	Sexes	Offspring number		Developmental duration (d)		Tibia length (mm)	
		First	Second	First	Second	First	Second
9	Female	15	16	20.13 ± 1.41	19.25 ± 1.13	0.33 ± 0.02a	0.29 ± 0.03b
	Male	7	9	19.29 ± 0.95	19.56 ± 1.59	0.33 ± 0.01a	0.27 ± 0.03b
12	Female	21	9	21.29 ± 1.65	19.44 ± 1.74	0.34 ± 0.02a	0.27 ± 0.03b
	Male	9	5	20.22 ± 2.11	20.20 ± 1.79	0.34 ± 0.03a	0.24 ± 0.03b
15	Female	44	2	19.00 ± 1.08	18.00 ± 1.41	0.35 ± 0.02	0.33 ± 0.01
	Male	24	0	18.67 ± 1.13	—	0.35 ± 0.02	—
ck	Female	37	—	20.14 ± 1.90	—	0.33 ± 0.02	
	Male	18	—	18.78 ± 1.70	—	0.31 ± 0.01	

**Fig 2:**
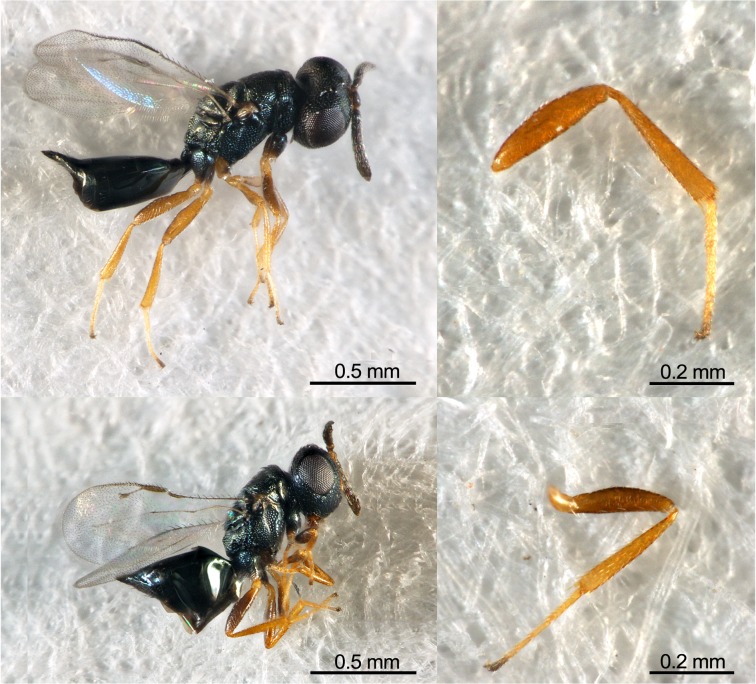
Two types of *P*. *vindemmiae* females and hind tibia. Upper and lower parasitoids are normal females and those from hyperparasitism, respectively. Offspring from hyperparasitism were smaller. Photograph by W Chen.

Hind tibia length of offspring differed among the control and the three treatments (*F*_2, 213_ = 119.16, *P* < 0.01) (Table 1). The tibia length of offspring from the second emerging event was significantly shorter than that from the first emerging event (*t*_49.94_ = 11.45, *P* < 0.01) (Fig 2), also was shorter than that from the control (*t*_57.66_ = 7.79, *P* < 0.01). When both sexes were analyzed separately (Table 2), the tibia length of female offspring differed among the control, the first and second emerging events (*F*_2, 141_ = 63.90, *P* < 0.01). And those from the second emerging event was shorter than that from the first emerging event (*t*_33.69_ = 8.71, *P* < 0.01), and also shorter than that from the control (*t*_38.77_ = 6.36, *P* < 0.01). The tibia length of male offspring differed among the control, the first and second emerging events (*F*_2, 69_ = 69.28, *P* < 0.01). And that from the second emerging event was shorter than that from the first one (*t*_16.18_ = 8.27, *P* < 0.01) and also shorter than that from the control (*t*_15.94_ = 5.22, *P* < 0.01).

Offspring number per group was significantly affected by the interaction of exposure interval and emerging event (*F*_1, 56_ = 42.96, *P* < 0.01), which suggested that the two factors were not independent. Offspring from the first emerging event enhanced with the increasing of exposure interval (*F*_1, 28_ = 27.23, *P* < 0.01), but those from the second emerging event reduced with the increasing interval (*F*_1, 28_ = 23.70, *P* < 0.01) (Table 1). When both sexes were analyzed separately (Table 2), with the increasing of exposure interval, both number of female (*F*_1, 28_ = 16.18, *P* < 0.01) and male offspring (*F*_1, 28_ = 11.55, *P* < 0.01) from the first emerging event enhanced (*F*_1, 28_ = 27.23, *P* < 0.01), but both number of female (*F*_1, 28_ = 11.51, *P* < 0.01) and male offspring (*F*_1, 28_ = 9.75, *P* < 0.01) from the second emerging event reduced (Table 2).

### Discrimination of the offspring from the first and second emerging events

**Fig 3:**
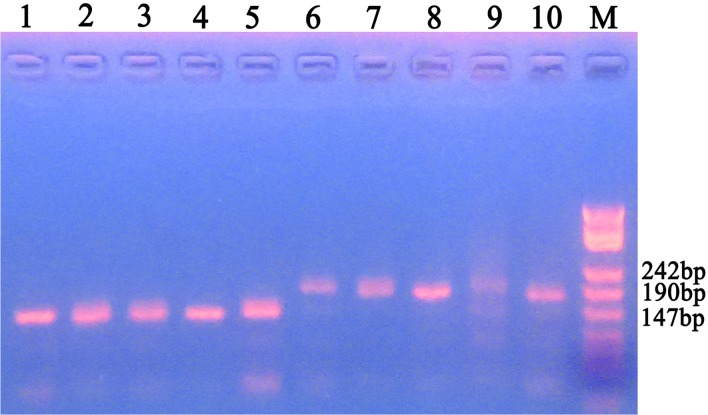
Electrophoresis of the primary and secondary *P*. *vindemmiae* females. 1–5: Wuhu strain, with about 150 bp fragments on gel electrophoresis, containing (AG)_10_ repeats. 6–10: Urumqi strain, with about 190 bp fragments, containing (AG)_28_ repeats.

**Fig 4:**
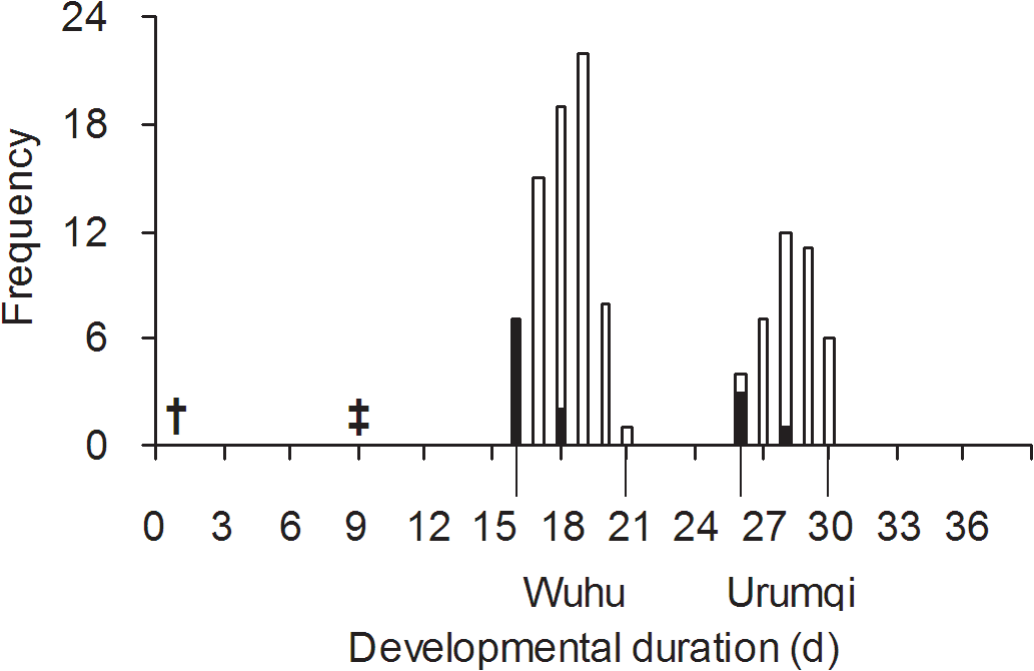
Developmental duration of *P*. *vindemmiae* from primary and secondary parasitism with a 9 day exposure interval. Wuhu and Urumqi strains represent the primary and secondary parasitoids, respectively. † and ‡ indicate the primary and secondary exposure, respectively. Open and solid bars indicate females and males, respectively.

The fragments migrated on the electrophoresis gel were about 150 bp for the Wuhu strain, while those for the Urumqi strain were about 190 bp (Fig 3). Nine days after the primary parasitism by the Wuhu strain *P*. *vindemmiae*, females of Urumqi strain were used as secondary parasitoids. Offspring also emerged at two obvious emerging events (Fig 4). All of the offspring in the first emerging event were from the primary parasitoids (*N* = 72), and all of those in the second one were from the secondary parasitoids (*N* = 40).

### Fitness of the primary and secondary foundresses’ offspring

Compared with *P*. *vindemmiae* females from primary parasitism, females from hyperparasitism used as foundresses had shorter longevity, fewer number of offspring (Table 3). Females from hyperparasitism also had lower values of degree of infestation (DI) and success rate of parasitism (SP) (Table 3), which showed that they had lower parasitism efficiency. When the two types of parasitoids encountered healthy *Drosophila* puparia, both male and female offspring were reduced in number for the foundresses from the hyperparasitism, but the offspring were similar in size (Table 4).

**Table 3:** Parameters of *P*. *vindemmiae* foundresses from the conspecific hyperparasitism and primary parasitism.

	Sample size	Longevity (d)	Number of offspring	DI	SP
Hyperparasitism	18	9.94 ± 2.80	12.72 ± 5.61	0.46 ± 0.24	0.43 ± 0.39
Primary parasitism	20	14.55 ± 6.45	55.74 ± 31.04	0.72 ± 0.27	0.55 ± 0.38
Significance		*F*_1, 36_ = 8.74**	*F*_1, 36_ = 53.72**	*F*_1, 376_ = 47.30**	*F*_1, 374_ = 8.55**

* represents *P* < 0.05

** represents *P* < 0.01

DI and SP represent the degree of infestation and the success rate of parasitism, respectively.

**Table 4:** Number and size of offspring of *P*. *vindemmiae* females from the conspecific hyperparasitism and primary parasitism.

	Number of females	Number of males	Female size	Male size
Hyperparasitism	10.83 ± 4.73	1.89 ± 1.37	0.35 ± 0.02	0.33 ± 0.02
Primary parasitism	41.35 ± 21.70	13.80 ± 10.76	0.35 ± 0.02	0.33 ± 0.03
Significance	*F*_1, 36_ = 51.09**	*F*_1, 36_ = 38.96**	*t*_291.10_ = 0.39, *P* = 0.69	*t*_36.65_ = 0.59, *P* = 0.58

** represents *P* < 0.01.

### Hyperparasitic behavior on infested hosts provided with healthy hosts

In the treatments with a healthy pupa proportion of 50%, 30%, and 10%, respectively, there were offspring from the secondary parasitoids (Urumqi strain) emerging out of the puparia parasitized by the primary parasitoids (Wuhu strain) (Table 5). With the decreasing of proportion of healthy hosts in the patch, the proportion of hyperparasitism offspring from the secondary parasitoid offspring was significantly affected (*F*_1, 16_ = 5.23, *P* = 0.02), which suggested a higher frequency of hyperparasitism when fewer healthy hosts.

**Table 5:** Offspring number emerging from healthy and infested hosts when exposed to *P*. *vindemmiae* females together.

Healthy: infested	Host number	Sample size	First emerging event (Wuhu strain)	Second emerging event (Urumqi strain)	
				Healthy	Infested
5:5	10	6	2.33 ± 0.52	2.17 ± 0.75	1.00 ± 0.00
3:7	10	8	3.00 ± 1.07	1.50 ± 0.93	1.13 ± 0.99
1:9	10	6	2.67 ± 1.51	0.17 ± 0.41	1.00 ± 0.63

## Discussion

**Fig 5:**
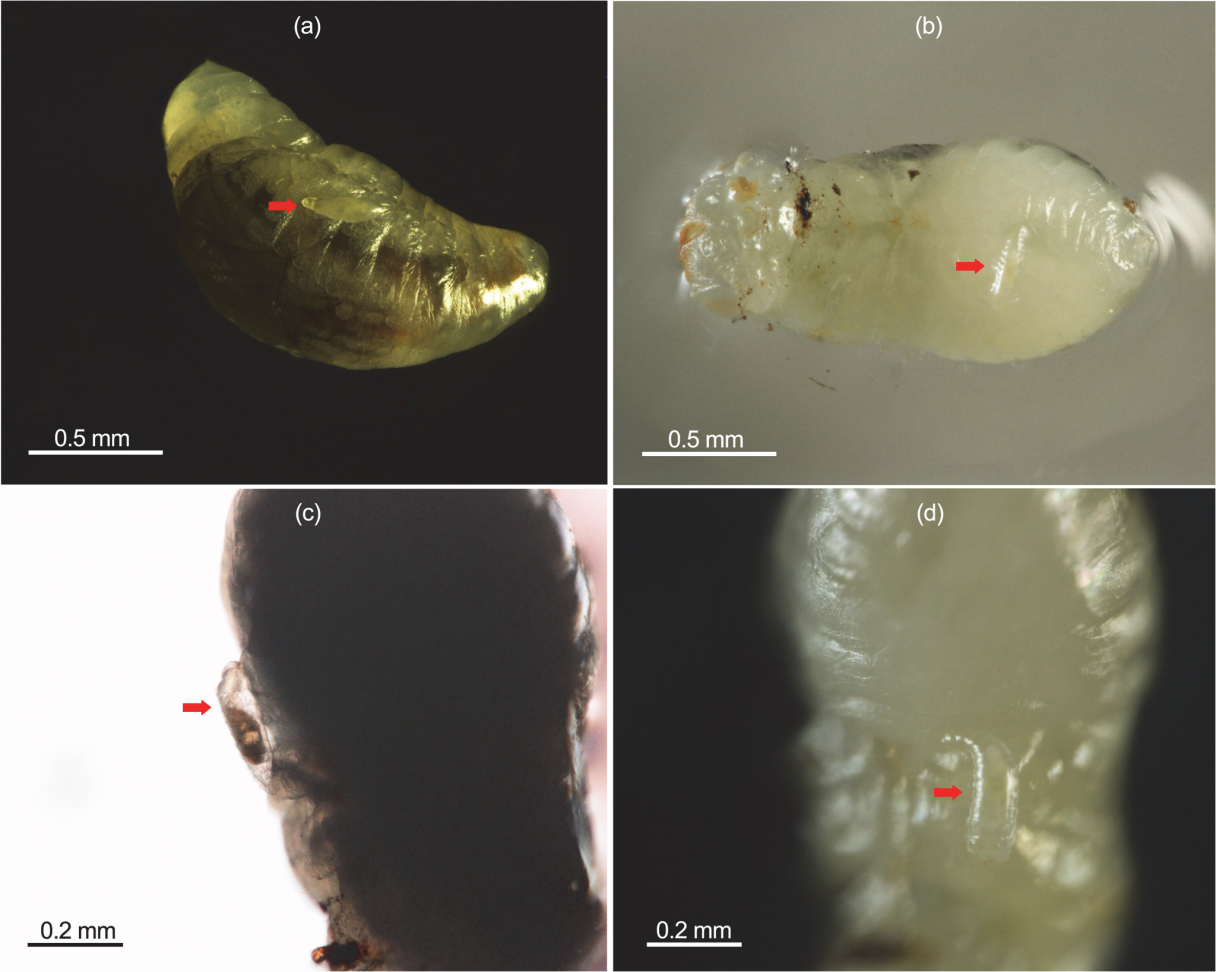
Eggs and larvae of secondary parasitoids on the prepupae of primary parasitoids. Eggs (a) were laid on the surface of the prepupae of primary parasitoids (infested nine days before). Larvae (b-d) fed on the primary conspecifics. Photograph by W Chen.

Our results showed that the duration of *P*. *vindemmiae* offspring emergence can be divided into two obvious events after *Drosophila* puparia have been parasitized twice with nine, twelve and fifteen day intervals; the offspring at the later emerging event probably belonged to the secondary foundresses. With two *P*. *vindemmiae* strains which can be identified by an SSR marker, we further confirmed that those emerging in the later event were from the secondary foundresses. Once the twice-parasitized *Drosophila* puparia had been dissected, we could see the eggs and larvae of the secondary foundresses on the surface of the prepupae or pupae of the primary foundresses (Fig 5). Those secondary larvae lived on the primary parasitoids and fed on them, which revealed a cannibalism behavior of larvae from the secondary foundresses on the primary conspecifics. Therefore, we thought that the secondary parasitism of *P*. *vindemmiae* on the prepupae or pupae of the primary foundresses in the *Drosophila* puparia could be considered conspecific hyperparasitism.

**Fig 6:**
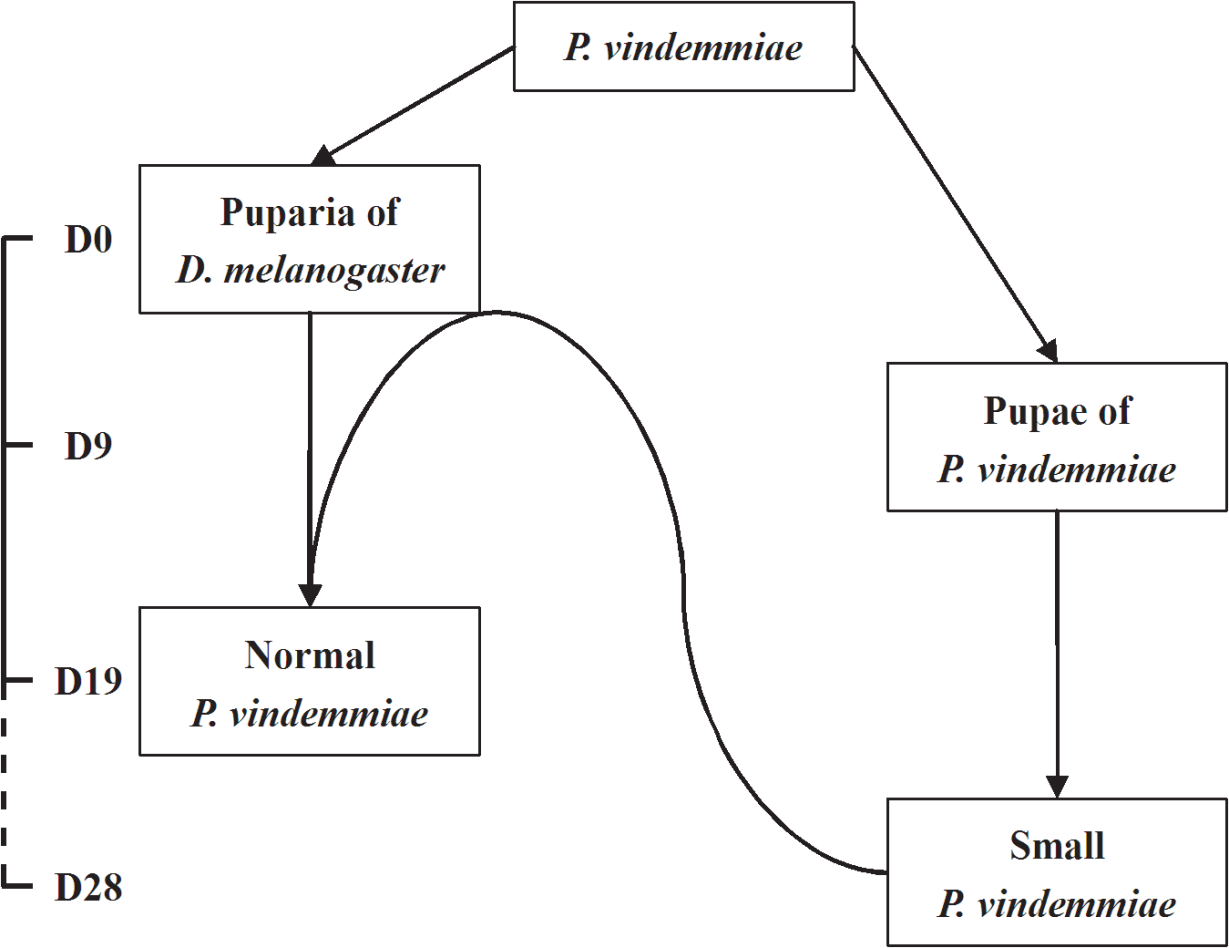
Facultative hyperparasitism of *P*. *vindemmiae* on conspecifics. D0-D28 indicate days after the primary parasitism. When healthy *Drosophila* puparia were lacking, those with primary conspecifics could be used and small parasitoid offspring occurred, but the emergence of the parasitoid delay for about nine days, and the risk of host limitation could be partly relieved. When healthy puparia were available, the smaller foundresses from the conspecific hyperparasitism laid eggs and normal progeny developed.

According to the morphological and developmental records [11,15], the offspring of the primary parasitoids would have reached the prepupal stage nine days after the parasitism. As mentioned above, the consumption of the *Drosophila* puparia would have been used up, and the nutrition of the secondary parasitoid’s larvae would be the primary conspecific prepuape or pupae. *P*. *vindemmiae* can use both *Drosophila* puparia and conspecifics, thus conspecific facultative hyperparasitism would fit the behavior of the parasitoid (Fig 6). From the *Drosophila* puparia parasitized twice with fewer than nine days as the interval, the offspring of the primary parasitoid would be in the larval phase at this point. Considering that the competition of *P*. *vindemmiae* larvae is body size dependent [23], the larvae of the secondary parasitoid would be smaller than the primary ones, and there might lose the competition in face of the primary ones. However, prepupae and pupae would be less aggressive [23], and thus could be used by the secondary larvae. When the hyperparasitism happened, we could not know whether the primary conspecifics were killed by the secondary larvae or by the secondary foundress when it lays in the puparium. There were offspring from the secondary parasitoids emerging out of the puparia infested by the primary parasitoids in the experiments, when healthy hosts were provided with a proportion of 50%, 30% and 10% in the oviposition patch (Table 5). The results suggest that the conspecific hyperparasitism behavior might not be a phenomenon only when healthy hosts are extreme lacking. But we had not proved the behavior in the field. And whether the conspecific hyperparasitism behavior was popular in natural environments needs further research.

Body size may be a key to understanding evolution of host species selection in ectoparasitoids, and has been considered to positively correlate with such fitness variables as life time fecundity, longevity, daily oviposition rate, offspring number, searching efficiency for hosts and so on [24–26]. *P*. *vindemmiae* is an idiobiont parasitoid, i.e. the development of hosts ceases when attacked; therefore, offspring size is strongly correlated with host size or nutrition [27]. Larval nutrition of parasitoid wasps is totally derived from the host, and the quality of the host affects parasitoid quality. With puparia of *D*. *melanogaster* as hosts, offspring of *P*. *vindemmiae* were smaller in size, with reduced longevity compared to when parasitizing other larger puparia of other species [28]. When superparasitize on a single host, the parasitoid would be forced to feed on the remains of the host after the consumption of the primary conspecifics, with reduced body size [11]. *P*. *vindemmiae* offspring are also reduced in size when hyperparasitism occurs with other primary tephritid fruit parasitoids, such as *F*. *arisanus*, *D*. *longicaudata*, *D*. *kraussii*, and *P*. *concolor*, compared to offspring from tephritid hosts *Bactrocera cucurbitae* (Coquillett) or *B*. *latifrons* (Hendel) [9]. *P*. *vindemmiae* (= *P*. *dubius* Ashmead) can also hyperparasitize *Trybliographa rapae* Westwood (Figitidae), with smaller offspring size as well [29]. When it facultatively hyperparasitized *A*. *tabida*, it reduced offspring sizes were observed [27]. *P*. *vindemmiae* is an idiobiont parasitoid, i.e. the development of hosts ceases when attacked; therefore, offspring size is strongly correlated with host size or nutrition [27]. Compared to healthy *Drosophila* puparia, those with parasitoid prepupae or pupae would have less nutrition because some parts of the primary conspecifics would be useless. The size of *P*. *vindemmiae* offspring was reduced when the hyperparasitism behavior occurred. Foundresses from the hyperparasitism had less fitness variables than those from primary parasitism, with shorter longevity, less life time fecundity, lower values of infestation degree, and lower success rate of parasitism, which was in line with theoretically prediction of the indicator effects of body size on parasitoid quality [24,26,30–32].

It seems that the hyperparasitism behavior would bring less fitness to foundresses, because it leads offspring reduced in size. However, the behavior would bring fitness variables to the *P*. *vindemmiae* population. The hosts of the parasitoid are mostly Cyclorrhapha flies, such as houseflies [13]. These hosts would be popular in some patches such as livestock farms, Aves nests and so on, according to the habitats of the flies [33–35]. Some hosts would form a refuge, which is difficult to find and makes the parasitoids less vulnerable [36]. Thus, it would be difficult for the parasitoid to find enough hosts sometimes. Our results also showed a higher frequency of hyperparasitism when fewer healthy hosts available (Table 5). To reproduce based on its own resources would provide the parasitoid more opportunity to sustain its population, considering it would make the emergence time prolonged for more than nine days in our experiment conditions (Fig 6). This behavior would also benefit the second parasitoid itself by removing others and occupying more resources for more offspring.

Hyperparasitism is usually thought to occur when a secondary female of another parasitoid wasp species lays her eggs on the host, and the larvae of the secondary species feed on the primary parasitoids [1]. Common hyperparasitism between species is known to be restricted to three taxa, i.e. Hymenoptera, Diptera, and Coleoptera (reviewed in [2]). Because some special cases of hyperparasitism such as heteronomous hyperparasitism can happen within certain species [3,4], the nutrition of the secondary parasitoids’ larvae would be the key point to distinguish this behavior from superparasitism and multiparasitism. We suggest that hyperparasitism happens when the larvae of a secondary parasitoid feed on the primary parasitoids.

Beyond for the species investigated in this study, some reports have shown that some parasitoids can feed on conspecific larvae or pupae. *Cephalonomia hyalinipennis* (Bethylidae) has been reported to lay eggs on conspecifically infested hosts, when there are no suitable primary hosts available, but the eggs die upon hatching [37]. *Dinarmus basalis* (Pteromalidae) can also lay eggs on hosts containing conspecific larvae or pupae, but very few *D*. *basalis* eggs from hyperparasitized conspecific old larvae reach the adult stage [38]. Also, some smaller offspring had been observed when *D*. *basalis* secondarily parasitized hosts, which was taken as a result of superparasitism and the depletion of host resources [38]. Whether these reports can be classified as conspecific hyperparasitism behavior needs further research.

Conspecific facultative hyperparasitism might be an extreme example of facultative hyperparasitism. Facultative hyperparasitism seems to be popular in parasitoid wasps, for example, the egg parasitoid *Ooencyrtus telenomicida* (Vassiliev) (Encyrtidae) can facultatively hyperparasitize *Trissolcus basalis* (Wollaston) (Platygastridae), another egg parasitoid of *Nezara viridula* (L.) (Heteroptera: Pentatomidae) [39]. Some idiobiont ectoparasitoids have a wide host range and are also frequently facultative hyperparasitoids [1,40]. *P*. *vindemmiae* is known as a polyphagous parasitoid, with a broad range of hosts [13,41], including some freeze-killed hosts [42,43]. Whether there is some conspecific hyperparasitism behavior in such polyphagous parasitoids would be interesting to determine.

The facultative hyperparasitism of *P*. *vindemmiae* differs from heteronomous hyperparasitism. Heteronomous hyperparasitism has been reported to occur when male larvae of some Aphelinidae (esp. Coccophaginae, a subfamily of Aphelinidae) attack a primary conspecific female larva [3–5]. In these heteronomous species, males have host relationships different from those of females, e.g. females are generally endoparasitoids of sternorrhynchous Hemiptera, such as scale insects, mealybugs, and whiteflies, but males may be hyperparasitoids, developing in or on conspecific females or other primary parasitoids [6,44,45]. According to host type, obligate autoparasitoids, facultative autoparasitoids, and alloparasitoids can be distinguished. Obligate autoparasitoids can only lay male eggs in hosts containing conspecific conspecifics, alloparasitoids always lay male eggs in heterospecific wasp hosts, and facultative autoparasitoids can lay male eggs in both hosts [3,6,45]. Only one sex (male) emerges from heteronomous hyperparasitism, and the male and female immature stages differ in terms of resource demands. However, both sexes of *P*. *vindemmiae* larvae can feed on conspecifics and emerge, without a sex tendency.

*P*. *vindemmiae* seems to be a Jack of all trades. When this species encounters healthy puparia, it is a primary parasitoid, and lays eggs on hosts from 13 families, including Anthomyiidae, Calliphoridae, Drosophilidae, Fanniidae, Lonchaeidae, Muscidae, Phoridae, Piophilidae, Sarcophagidae, Stratiomyidae, Tachinidae, Tephritidae, and Cecidomyidae [13]. When it encounters an infested host, it can be a hyperparasitoid, with hosts including Braconidae, Diapriidae, Encyrtidae, Eucoilidae, and Pteromalidae [13]. Primary parasitoids of species such as *A*. *tabida* can also be the hosts of *P*. *vindemmiae* [8–10]. Superparasitism also fits the parasitoid, as several conspecific eggs were common during the dissection of infested hosts, though usually only one offspring would emerge [15,23,46]. The larvae of *P*. *vindemmiae* would also compete with the primary larvae of other species for host resources, which indicates multiparasitism [9]. *P*. *vindemmiae* is widely distributed throughout the world (including Asia, North and South America, Africa, Europe, and Australia) [13]. Even in the newly invasive spotted wing drosophila, *D*. *suzukii*, *P*. *vindemmiae* was the first and only parasitoid found in American orchards [19,47], and can successfully parasitize the pest in laboratory experiments [41]. It is also widely distributed on the Chinese mainland (unpublished data). It has been observed to have aggressive behavior and fight with other parasitoids [23,46], and would have excellent abilities compared to other species in the same ecological niche. The behavior of conspecific hyperparasitism is one of these abilities, considering that the parasitoid can sustain its population for almost one generation with only its conspecific population. The interspecies interaction of the parasitoid with other parasitoids is also interesting and it would be helpful to further use this species for the biological control of pest insects such as spotted wing drosophila.
